# Regulation of Exogenous Strigolactone on Storage Substance Metabolism and Endogenous Hormone Levels in the Early Germination Stage of Rice Seeds Under Salt Stress

**DOI:** 10.3390/antiox14010022

**Published:** 2024-12-27

**Authors:** Jianqin Zhang, Dianfeng Zheng, Naijie Feng, Aaqil Khan, Rui Deng, Jian Xiong, Linchong Ding, Zhiyuan Sun, Jiahuan Li, Xiaohui Yang, Chen Wu

**Affiliations:** 1 College of Coastal Agriculture Sciences, Guangdong Ocean University, Zhanjiang 524088, China; 2 South China Center of National Saline-Tolerant Rice Technology Innovation Center, Zhanjiang 524088, China

**Keywords:** GR24, rice, seed germination, metabolism of stored substances, endogenous hormones

## Abstract

Salt stress inhibits rice seed germination. Strigolactone (GR24) plays a vital role in enhancing plant tolerance against salt stress. However, GR24’s impact on the metabolism of stored substances and endogenous hormones remains unclear. This study investigated the impact of exogenous GR24 on the metabolism of stored substances and endogenous hormones during the early stages of rice seed germination under salt stress. The results showed that salt stress significantly reduced the germination rate, germination potential, germination index, radicle length, germ length, and fresh and dry weights of the radicle and germ under salt stress. Pre-treatment (1.2 μmol L^−1^ GR24) significantly reduced the inhibition of salt stress on rice seed germination and seedling growth. GR24 promoted the decomposition of starch by enhancing the activities of α-amylase, β-amylase, and total amylase and improved the levels of soluble sugars and proteins and the conversion rate of substances under salt stress. GR24 effectively enhanced the activities of superoxide dismutase (SOD), catalase (CAT), and ascorbate peroxidase (APX); increased ascorbic acid (ASA) and glutathione (GSH) levels; and reduced malondialdehyde (MDA) content. This reduced the oxidative damage of salt stress. Furthermore, GR24 significantly increased the contents of strigolactones (SLs), auxin (IAA), gibberellin (GA3), cytokinin (CTK) as well as IAA/ABA, CTK/ABA, GA/ABA, and SL/ABA ratios and reduced abscisic acid (ABA) levels. The current findings indicate that GR24 effectively mitigates the adverse impact salt stress by regulating antioxidant enzyme activity and endogenous hormone balance.

## 1. Introduction

Global climate change and inadequate irrigation practices are the major issues to increase soil salinization [1]. The accumulation of salt in the soil restricts the cultivated area available for crops. Soil salinity inhibits seed germination [2] and seedling survival [3], which in turn results in a reduction in crop productivity and quality [4]. The initial impact of salt stress on seed germination is through osmotic and ion toxicity, or a combination of both [5]. Seed germination is a critical stage in the seedling establishment process [6], and it is also a complex process that is most susceptible to adverse environmental conditions, especially salt stress [7]. Seed germination involves aspects such as water absorption, degradation of stored substances, and energy metabolism for seed respiration [8]. The presence of high levels of salinity in the soil can result in a reduction of soil water potential, thereby impairing the seed’s ability to absorb water. This, in turn, can lead to an inhibition of both embryonic development and the formation of new organs [9]. Salt stress has also been demonstrated to affect physiological and metabolic processes within seeds. These effects include alterations in the activity of antioxidant enzymes, inhibition of the metabolism and respiration of stored substances, and the production of detrimental compounds, which can impede seed germination and seedling growth [10,11]. It is of considerable importance, therefore, to seek ways of mitigating the adverse effects of salt stress on the germination and early development of seeds.

Rice (*Oryza sativa* L.) is a vital and dietary staple for two-thirds of China’s population [12]. It is therefore imperative to maintain a consistent increase in rice production in order to guarantee social stability and development [13]. The early stages of rice cultivation, including germination, emergence, and seedling formation, are the important traits due to their role in determining the overall plant growth of the rice crop [14]. However, the growth of rice is threatened by salt stress as a consequence of the increasing salinization of the soil [15]. The presence of salt in the soil during field seeding has been observed to impede the germination and emergence of seeds and induce irregularities in the emergence of rice [14]. In the context of agricultural production, a number of factors, both endogenous and exogenous, play a role in the germination and subsequent growth of seeds [16]. Exogenous plant growth regulators have been demonstrated to facilitate germination and enhance seed metabolism, thereby improving seedling emergence and growth under disparate environmental circumstances [7,17].

Strigolactones (SLs) are a new type of plant hormone that were first discovered as compounds that induce seed germination in root parasitic plants [18]. GR24 is one of the analogues of synthetic SL. A large number of studies have reported that GR24 is involved in the growth and development of plants and responds to various environmental stresses, including salt stress [19,20], drought stress [21], low-temperature stress [22], heavy metal stress [23], and low-light stress [24]. Previous studies revealed that the application of exogenous GR24 can improve the germination ability of plant seeds under different environmental stresses by regulating various defense mechanisms and plant cell metabolism before or during stress [25]. Li et al. [25] found that exogenous GR24 pre-treatment alleviated the inhibition of cucumber seed germination induced by salt stress by enhancing antioxidant capacity. Bradow et al. [26] showed that strigolactone at the right concentration could promote and stimulate germination in dormant seeds of *Striga* and *Lactuca*. Al-Amri et al. [27] demonstrated that GR24 induces germination in tomato seeds and enhances subsequent growth performance. Toh et al. [28] found that exogenous GR24 pre-treatment could promote seed germination. It is concluded that the application of GR24 from an external source has the potential to enhance the germination and subsequent development of plants subjected to stress conditions.

Currently, there are limited data on GR24 soaking to alleviate salt stress and its inhibition of rice seed germination. The aim of the study was to (1) identify the optimal concentration of exogenous GR24 for soaking seeds prior to germination and (2) investigate the impact of exogenous GR24 on the metabolism of stored substances, antioxidant capacity, and hormone levels throughout the germination of rice seeds under salt stress. This study will further insights into the physiological processes underlying the salt tolerance induced by GR24 in plants.

## 2. Materials and Methods

### 2.1. Test Materials

The experiment was conducted in 2024 in the Laboratory of Seawater Rice Physiology and Biochemistry, Binhai Agricultural College, Guangdong Ocean University. The rice variety Huang Huazhan was selected, as provided by Hunan Jinfeng Seed Industry Co., Ltd. (Hunan, China). The test agent was the strigolactone synthetic analogue GR24.

### 2.2. Experimental Design

#### 2.2.1. Concentration Screening Test Design

Fully developed, neat, and healthy Huang Huazhan seeds were selected, disinfected with 3% hydrogen peroxide for 15 min, and rinsed repeatedly with ultrapure water for 5 times. The seeds were soaked in GR24 solution at different concentrations (0.3, 0.6, 1.2, 2.4, and 4.8 μmol L^−1^) and soaked at 30 °C for 24 h. The control was soaked in clean water. Three layers of filter paper were placed in the germination box and wet with 20 mL of water or 80 mmol L^−1^ NaCl solution. Seeds of uniform color and size were evenly arranged, sown in the germination box, and placed in a constant temperature incubator to culture. First, to prevent light from burning the bud points, seeds were incubated in the dark for 48 h. Then, seeds were exposed to light and darkness for 12 h each. The temperature was 30/28 °C (12/12 h), and the humidity was 75%. The light was 15,000 Lx, and the incubation time was 192 h. In order to prevent the loss of water from the germination box, a quantitative supplement of 5 mL of water was made every day, and the filter paper was changed once every three days. Six treatments were established: CK, water soaking + water treatment; T1, 0.3 μmol L^−1^ GR24 soaking + water treatment; T2, 0.6 μmol L^−1^ GR24 seed soaking + water treatment; T3, 1.2 μmol L^−1^ GR24 seed soaking + water treatment; T4, 2.4 μmol L^−1^ GR24 soaking + water treatment; and T5, 4.8 μmol L^−1^ GR24 soaking + water treatment. There were four replicates per treatment, with 100 capsules per replicate. Root and shoot length were measured at 96, 144, and 192 h, and 20 plants were included in each replicate. According to the above indicators, the most suitable strigolactone concentration was screened.

#### 2.2.2. Experimental Design of Exogenous GR24 Soaking for Seed Germination Under Salt Stress

Based on the previous concentration screening test results, 1.2 μmol L^−1^ strigolactone was selected as the appropriate test concentration. The germination test method is the same as noted in Section 2.2.1. Four treatments were established: (1) clear water + clean water treatment, (2) 1.2 μmol L^−1^ GR24 seed soaking + clean water treatment (G), (3) water soaking + 80 mmol L^−1^ NaCl treatment (N), and (4) 1.2 μmol L^−1^ GR24 soaking + 80 mmol L^−1^ NaCl treatment (NG). Samples of seeds, radicles, and germs were taken at 24 h, 36 h, 48 h, 72 h, 96 h, 144 h, and 192 h and stored at −40 °C.

## 3. Measurement Items and Methods

### 3.1. Determination of Germination Rate, Germination Potential, and Germination Index

Regarding the germination standard, the germination length of rice seeds was equal to half of the seed length, and the number of seed germinations was recorded every day. The seed germination potential was counted at 96 h, and the seed germination rate was determined at 192 h.

Germination potential = number of seeds germinated within a specified number of days/number of seeds tested × 100%.

Germination rate = n/N × 100%, where n is the number of germinations, and N is the total number of seeds.

Germination index = ∑Gt/Dt, where Gt is the number of seeds germinated on day t, and Dt is the corresponding number of germination days.

### 3.2. Seed Germination Morphology and Dry Weight

The root and shoot lengths of each treatment were measured at 96, 144, and 192 h, and the fresh weight and dry weight were measured at 192 h with an electronic balance. The number of lateral roots was counted to calculate the substance conversion rate. Here, 20 plants were used for each replicate.

Substance conversion rate (%) = [(radicle dry weight + germ dry weight)/(seed residue dry weight + radicle dry weight + germ dry weight)] × 100%.

### 3.3. Determination of Total Amylase, α-Amylase, and β-Amylase Activities in Seeds

Total amylase, α-amylase, and β-amylase activities were determined according to the method of Dai et al. [29] with slight modifications. The homogenate was obtained by grinding in pH 5.6 citric acid buffer and transferred to a centrifuge tube. The sample was extracted in a 40 °C water bath for 1 h and cool to room temperature after the water bath. The supernatant obtained after centrifugation at 3000 rpm for 10 min was the amylase stock solution. OD values were measured at 520 nm. According to the formula, total amylase, α-amylase, and β-amylase activities are calculated.

### 3.4. Determination of Starch and Soluble Sugar Content in Seeds

The starch and soluble sugar contents were obtained based on the method of Du et al. [30] with slight modifications. First, the seeds were ground in 80% ethanol, and the homogenate was placed into a test tube. The tube was placed in a water bath at 80 °C for 20 min and centrifuged for 5 min (4000 rpm). Then, the supernatant was collected for the determination of the soluble sugar content, and the remaining residue was used for the determination of the starch content. Finally, the absorbance values were colorimetrically determined at a wavelength of 620 nm.

### 3.5. Determination of Soluble Protein Content in Seeds

The soluble protein content was determined using the Coomassie brilliant blue G-250 staining method described by Luo et al. [31]. The OD value was determined at a wavelength of 595 nm.

### 3.6. Determination of Antioxidant Enzyme Activity in Seeds

Superoxide dismutase (SOD) was determined using the nitrogen blue tetrazolium (NBT) method described by James et al. [32]. Peroxidase (POD) was determined as described by Nakano et al. [33]. Catalase [9] was determined based on the amount of H_2_O_2_ reduction per unit time according to the method described by Ekinci et al. [34]. Ascorbate peroxidase (APX) is measured using the amount of ASA oxidized per unit time [33].

### 3.7. Determination of the Antioxidant Content in Seeds

The ascorbic acid (ASA) content was determined according to the method described by Kampfenke and Costa [32], and glutathione (GSH) content was determined according to the method described by Tyburski and Tretyn [35]. Absorbance values were measured at 534 nm and 412 nm, respectively.

### 3.8. Determination of MDA Content in Seeds

The MDA content was determined using the thiobarbituric acid (TBA) method [36].

### 3.9. Determination of the Endogenous Hormone Content in the Radicle and Germ

The content of endogenous hormones was measured by Shanghai Enzyme-linked Biotechnology Co., Ltd. (Shanghai, China). A two-antibody one-step sandwich enzyme-linked immunosorbent assay (ELISA) kit (Shanghai Enzyme-linked Biotechnology Co., Ltd. Shanghai, China) was used. The absorbance (OD value) of the enzyme marker was measured at a wavelength of 450 nm, and the concentration of the sample was calculated.

### 3.10. Statistical Methods

All data were processed using Microsoft Excel 2020. ANOVA was performed using SPSS 20.0, and multiple comparisons were performed using Duncan’s multiple comparison test. The analysis results were expressed as the mean and standard error, with significant differences indicated by different lowercase letters (*p* < 0.05). The diagram in this article was created using Origin 2021 (Version number: 6.17, website link: https://www.originlab.com/ and date of access: 15 April 2024).

## 4. Results

### 4.1. GR24 Concentration Screening

**Figure 1:**
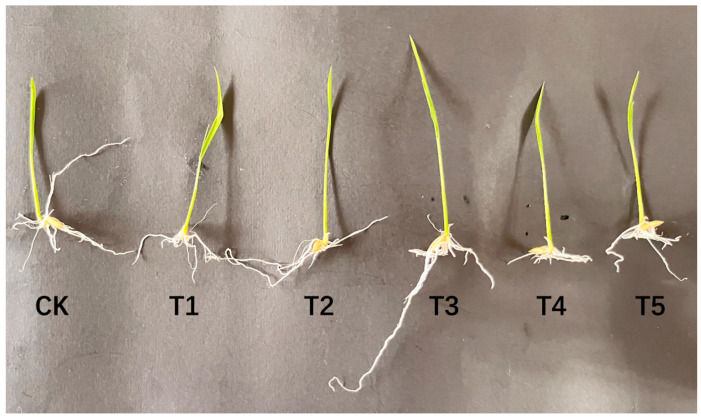
Phenotypic images of rice radicle length and plumule length at different concentrations of GR24 imbibition (192 h).

After soaking in different concentrations of GR24, root length and shoot length increased first and then decreased. The peak radicle length and plumule length were obtained with 1.2 μmol L^−1^ GR24 (Table 1 and Figure 1), and follow-up experiments were carried out using 1.2 μmol L^−1^ GR24 as the optimal concentration for soaking.

### 4.2. Effect of Exogenous GR24 Soaking on the Germination Traits of Rice Seeds Under Salt Stress

Salt stress significantly inhibited the germination index of rice seeds, while GR24 soaking treatment effectively alleviated the inhibition of seed germination under salt stress (Table 2). Compared with CK, salt stress significantly reduced seed germination potential, germination rate, and germination index by 42.15%, 16.46%, and 19.7%, respectively. Compared with salt stress, GR24 soaking treatment significantly increased seed germination potential, germination rate, and germination index by 56.08%, 18.58%, and 15.67%, respectively, under salt stress.

### 4.3. Effect of Exogenous GR24 Soaking on Germ Length and Radicle Length Under Salt Stress

**Figure 2:**
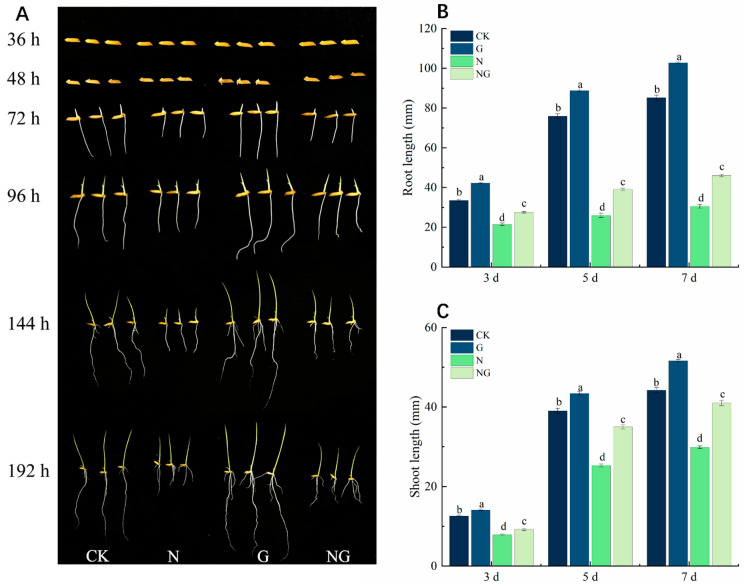
Effect of exogenous GR24 seed soaking on radicle length and germ length under salt stress. (**A**) Phenotype of germination of rice seeds at 36–192 h under different treatments; (**B**) Radicle length; (**C**) Germ length. CK: Clear water + clear water treatment; G: 1.2 µmol L^−1^ GR24 dipped seed + clear water treatment; N: clear water dipped seed + 80 mmol L^−1^ NaCl treatment; NG: 1.2 µmol L^−1^ GR24 dipped seed + 80 mmol L^−1^ NaCl treatment. Values are means ± SD of three replicates. Different letters in the data columns indicate significant differences (*p* < 0.05) according to Duncan’s test.

Salt stress significantly inhibited the radicle length and germ length, while the application of exogenous GR24 soaking treatment effectively alleviated the inhibition on the radicle and germ under salt stress (Figure 2). Compared to CK, NaCl stress significantly reduced the germ length on the third, fifth, and seventh days by 37.61%, 34.13%, and 32.55%, respectively. Compared to CK, salt stress significantly reduced the radicle length on the third, fifth, and seventh days by 35.98%, 68.68%, and 66.45%, respectively. Compared to NaCl stress, exogenous GR24 soaking treatment significantly increased germ length and radicle length on the third, fourth, fifth, and seventh days by 17.01%, 23.44%, 17.19%, 25.05%, 50.40%, and 43.39%, respectively.

### 4.4. Effect of Exogenous GR24 Soaking on Dry Weight, Fresh Weight, and Lateral Root Number of Radicles Under Salt Stress

Salt stress significantly inhibited rice seed germination growth indexes while the application of exogenous GR24 soaking treatment increased the morphological indexes of rice seed germination under NaCl stress (Table 3). Compared to CK, salt treatment significantly reduced the germ fresh weight, radicle fresh weight, germ dry weight, radicle dry weight, and lateral root count, with average decreases of 36.59%, 31.61%, 30.66%, 29.58%, and 36.59%, respectively. The application of GR24 soaking treatment significantly increased the germ fresh weight, radicle fresh weight, dry weight of germ, dry weight of radicle, and several lateral roots, with average increases of 9.89%, 32.05%, 39.84%, 36.00%, and 50.00%, respectively under salt stress. These results indicated that GR24 seed soaking could effectively alleviate the inhibition of salt stress on rice seed germination.

### 4.5. Effect of Exogenous GR24 Soaking on the Substance Conversion Rate During Seed Germination Under Salt Stress

**Figure 3:**
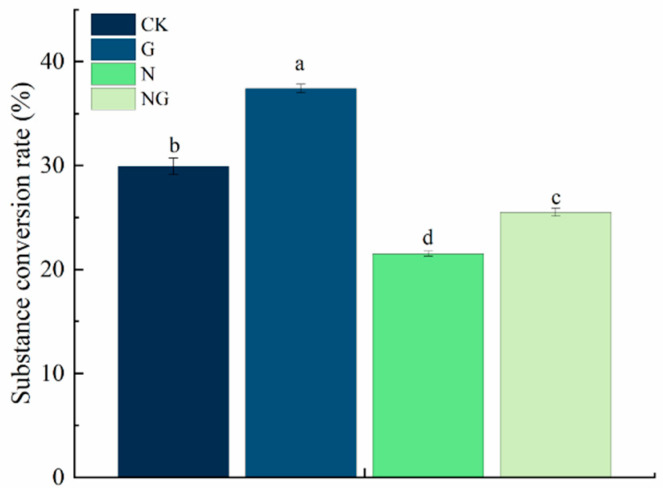
Effect of exogenous GR24 soaking on the conversion rate of seeds during seed germination under salt stress (192 h). CK: Clear water + clear water treatment; G: 1.2 µmol L^−1^ GR24 dipped seed + clear water treatment; N: clear water dipped seed + 80 mmol L^−1^ NaCl treatment; NG: 1.2 µmol L^−1^ GR24 dipped seed + 80 mmol L^−1^ NaCl treatment. Values are means ± SD of three replicates. Different letters in the data columns indicate significant differences (*p* < 0.05) according to Duncan’s test.

Salt stress significantly inhibited the substance conversion rate of rice seeds during germination, while exogenous GR24 soaking treatment alleviated the impact of salt stress (Figure 3). Compared to CK, the substance conversion rate under salt treatment was significantly reduced (28.16%). Under salt stress, the substance conversion rate of exogenous GR24 soaking treatment was significantly increased (18.58%). These results indicated that GR24 seed soaking could effectively improve the decomposition and utilization of stored substances during rice seed germination under salt stress.

### 4.6. Effects of Exogenous GR24 Soaking on Soluble Protein and Soluble Sugar Content During Seed Germination Under Salt Stress

**Figure 4:**
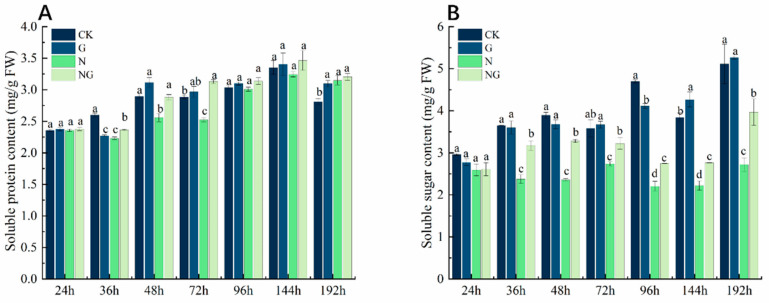
Effects of exogenous GR24 soaking on the contents of (**A**) soluble protein and (**B**) soluble sugar during seed germination under salt stress. CK: Clear water + clear water treatment; G: 1.2 µmol L^−1^ GR24 dipped seed + clear water treatment; N: clear water dipped seed + 80 mmol L^−1^ NaCl treatment; NG: 1.2 µmol L^−1^ GR24 dipped seed + 80 mmol L^−1^ NaCl treatment. Values are means ± SD of three replicates. Different letters in the data columns indicate significant differences (*p* < 0.05) according to Duncan’s test.

As shown in Figure 4A, the soluble protein content showed an overall upward trend during seed germination. Exogenous GR24 had a significant effect on the soluble protein content in seeds under salt stress (*p* < 0.05). Compared to CK, the soluble protein content in rice seeds decreased significantly by 14.66% on average under salt stress from 36 h to 72 h and also decreased under salt stress at 96–192 h, but the difference was not significant. After exogenous GR24 soaking treatment, the soluble protein increased to varying degrees with the extension of salt stress time. Compared to salt stress, the soluble protein content in rice seeds increased by 19.32% on average after exogenous GR24 soaking under salt stress conditions. The soluble sugar content showed an overall upward trend, and salt stress significantly reduced the soluble sugar content in rice seeds (Figure 4B). Compared to CK, the soluble sugar in rice seeds under salt stress was significantly reduced by 70.61% on average, and the maximum decrease was 112.96% at 96 h. Exogenous GR24 soaking treatment significantly increased the soluble sugar content in rice seeds under salt stress. Compared to salt stress, the soluble sugar content in rice seeds increased by 23.19% on average after exogenous GR24 soaking under salt stress.

### 4.7. Effect of Exogenous GR24 Soaking on Starch Content During Seed Germination Under Salt Stress

**Figure 5:**
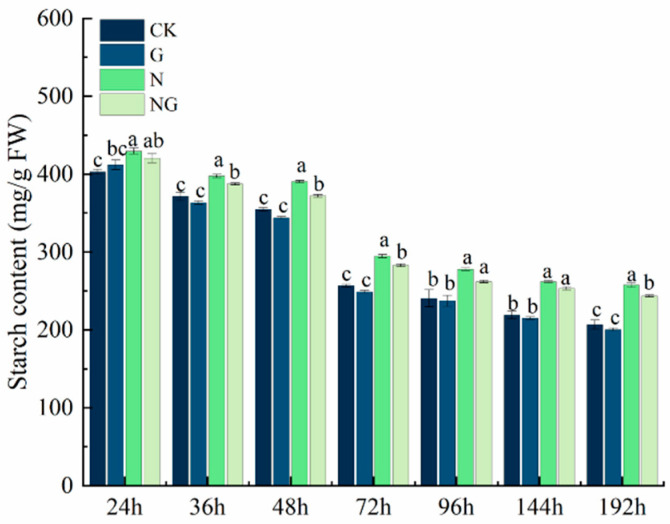
Effect of exogenous GR24 seed soaking on starch content during seed germination under salt stress. CK: Clear water + clear water treatment; G: 1.2 µmol L^−1^ GR24 dipped seed + clear water treatment; N: clear water dipped seed + 80 mmol L^−1^ NaCl treatment; NG: 1.2 µmol L^−1^ GR24 dipped seed + 80 mmol L^−1^ NaCl treatment. Values are means ± SD of three replicates. Different letters in the data columns indicate significant differences (*p* < 0.05) according to Duncan’s test.

The changes in starch content under salt stress are shown in Figure 5. The starch content gradually decreased during seed germination, and the starch content was higher than that of CK under salt stress. Compared to CK, the starch content in rice seeds under salt stress was significantly higher by 12.04% on average. Exogenous GR24 soaking increased the decomposition ability of starch under salt stress. Compared to salt stress alone, the starch content of exogenous GR24 soaking under salt stress was significantly reduced by 2.58%, 4.97%, 4.15%, and 5.82% at 36 h, 48 h, 72 h, and 192 h, respectively, and decreased by 6.05% and 3.39% at 96 h and 144 h, respectively. These results showed that GR24 could effectively alleviate the toxic effect of salt stress on rice seed germination by increasing amylase activity and accelerating starch decomposition.

### 4.8. Effect of Exogenous GR24 Soaking on Amylase Activity During Seed Germination Under Salt Stress

**Figure 6:**
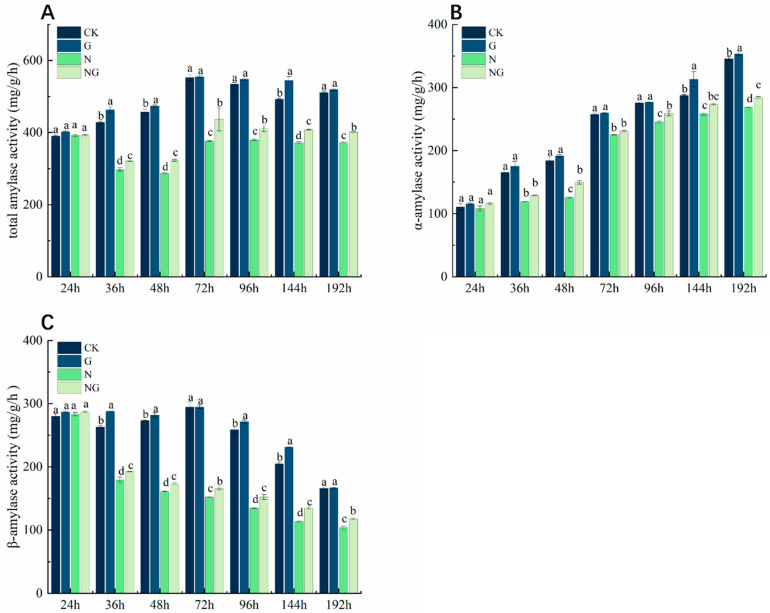
Effects of exogenous GR24 soaking on (**A**) total amylase activity, (**B**) α-amylase activity, and (**C**) β-amylase activity during seed germination under salt stress. CK: Clear water + clear water treatment; G: 1.2 µmol L^−1^ GR24 dipped seed + clear water treatment; N: clear water dipped seed + 80 mmol L^−1^ NaCl treatment; NG: 1.2 µmol L^−1^ GR24 dipped seed + 80 mmol L^−1^ NaCl treatment. Values are means ± SD of three replicates. Different letters in the data columns indicate significant differences (*p* < 0.05) according to Duncan’s test.

Salt stress severely inhibited total amylase activity, α-amylase activity, and β-amylase activity during seed germination (Figure 6A–C). α-Amylase activity showed an upward trend with the increase in germination time (Figure 6B), while β-amylase activity gradually decreased (Figure 6C). Compared to CK, the total amylase activity, α-amylase activity, and β-amylase activity were significantly decreased by 41.37%, 25.37%, and 69.61% on average from 36 h to 192 h. Exogenous GR24 soaking could effectively increase the activities of total amylase, α-amylase, and β-amylase under salt stress. Compared to salt stress, the total amylase activity and β-amylase activity in seeds were significantly increased by 11.24% and 12.97%, respectively. The activity of α-amylase was significantly increased by 15.81%, 5.18%, and 5.84% at 48 h, 96 h, and 192 h, respectively.

### 4.9. Effect of Exogenous GR24 Soaking on Antioxidant Enzyme Metabolism During Seed Germination Under Salt Stress

**Figure 7:**
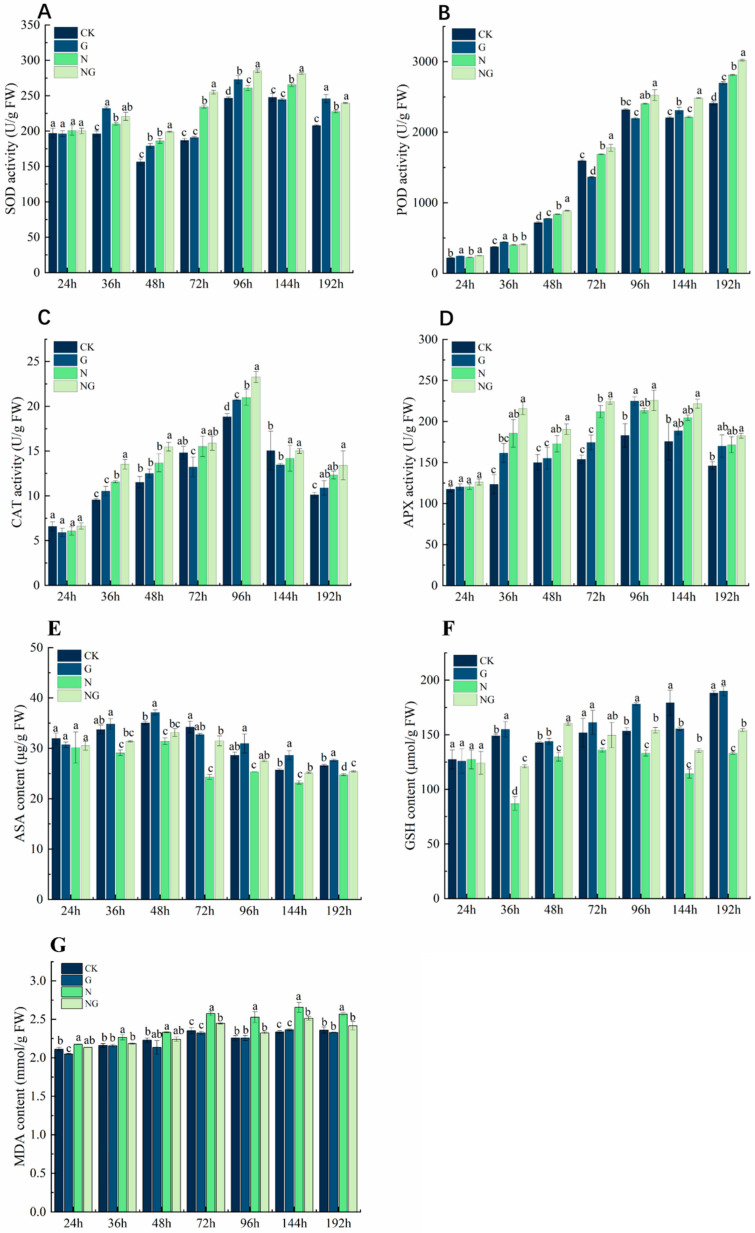
Effect of exogenous GR24 infusion on antioxidant metabolism during seed germination under salt stress. (**A**) SOD activity; (**B**) POD activity; (**C**) CAT activity; (**D**) APX activity; (**E**) ASA content; (**F**) GSH content; (**G**) MDA content. CK: Clear water + clear water treatment; G: 1.2 µmol L^−1^ GR24 dipped seed + clear water treatment; N: clear water dipped seed + 80 mmol L^−1^ NaCl treatment; NG: 1.2 µmol L^−1^ GR24 dipped seed + 80 mmol L^−1^ NaCl treatment. Values are means ± SD of three replicates. Different letters in the data columns indicate significant differences (*p* < 0.05) according to Duncan’s test.

The changes in antioxidant enzyme activity in rice seeds under salt stress are shown in Figure 7. Under salt stress, the activities of SOD, CAT, and APX in rice seeds increased first and then decreased, and the POD activity gradually increased (Figure 7A–D). The activities of SOD, CAT, and APX in rice seeds reached their maximum values at 96 h under salt stress. Compared to CK, salt stress significantly increased SOD, POD, CAT, and APX activities in rice seeds by 15.8–20.12%, 6.39–14.31%, 15.84–34.60%, and 14.27–33.46% at 36–192 h, respectively. Exogenous GR24 imbibition treatment further increased SOD, POD, CAT, and APX activities in rice seeds under salt stress (Figure 7A–D). SOD activity was significantly increased by 4.85–8.59% (Figure 7A), POD activity by 5.47–15.30% (Figure 7B), and CAT and APX activities by 11.56–20.28% and 9.28–14.04% (Figure 7C,D), respectively, in the seeds after application of GR24 soaking treatments compared to salt stress treatments.

During seed germination, the content of ASA was significantly reduced under salt stress (Figure 7E). The content of ASA was significantly reduced by 16.6% on average under salt stress compared to CK. Exogenous GR24 immersion treatment increased ASA content during seed germination. Compared with salt stress, the exogenous GR24 soaking treatment under salt stress significantly increased ASA content by an average of 9.4% from 72 h to 196 h of germination. There was also an increase in ASA content from 24 h to 48 h, but the difference was not significant. GSH content showed an increasing and then decreasing trend under salt stress, and seeds were affected by salt stress during germination. Overall, the GSH content was lower than CK (Figure 7F). The GSH content of rice seeds under salt stress was significantly reduced by 11.7–71.2% compared to CK. Exogenous GR24 increased GSH content. GSH content was significantly increased by 19.0–28.2% in seeds treated with exogenous GR24 infusion under salt stress compared to salt stress. These results indicate that exogenous GR24 imbibition treatment increased ASA and GSH contents under salt stress.

With the extension of salt stress exposure time, the MDA content gradually increased under salt stress treatment (Figure 7G). Compared to CK, the MDA content in rice seeds increased significantly by 7.6% on average under salt stress, and the MDA content increased significantly from the 72 h. Exogenous GR24 soaking treatment can effectively reduce the content of MDA. Compared to salt stress, the MDA content in rice seeds was significantly reduced by 5.2% on average after exogenous GR24 soaking under salt stress. These results indicated that exogenous GR24 could significantly reduce membrane lipid peroxidation in rice seeds under salt stress.

### 4.10. Effect of Exogenous GR24 Soaking on Endogenous Hormone Content in the Radicle and Germ Under Salt Stress

The endogenous hormone levels of the radicle and germ under salt stress are shown in Table 4. Salt stress decreased the content of SLs in the radicle, but increased the content of SLs in the germ. Compared to CK, the content of SLs in the radicle was significantly reduced by 42.09% under salt stress. The content of SLs in the germ was significantly increased by 4.33%. Exogenous GR24 soaking treatment increased the accumulation of SLs in the radicle and germ under salt stress. Compared to salt stress, the contents of SLs in the radicle and germ treated with exogenous GR24 under salt stress were significantly increased by 61.15% and 64.2%, respectively. Salt stress increased the content of endogenous ABA in the radicle and germ, while exogenous GR24 decreased the content of ABA in radicle and germ. Compared to CK, the content of ABA in radicle and germ under salt stress was significantly increased by 36.95% and 41.93%, respectively. Compared to salt stress, the ABA content in the radicle and germ was significantly reduced by 70.11% and 48.47%, respectively, after exogenous GR24 seed soaking treatment under salt stress, and even the ABA content in radicle was lower than that of the control. Salt stress significantly inhibited the contents of endogenous IAA, GA3, and CTK in the radicle and germ (Table 4). Compared to CK, the IAA content in radicle and germ under salt stress was significantly reduced by 24.27% and 4.16%. Compared to CK, the contents of GA3 in radicle and germ were significantly reduced by 30.14% and 27.39%, respectively, under salt stress. The content of CTK was significantly reduced by 95.76% and 57.85%, respectively. In contrast, exogenous GR24 increased the content of endogenous IAA, GA3, and CTK. Compared to salt stress, the IAA content in radicle and germ increased significantly by 18.29% and 28.31%, respectively, after exogenous GR24 soaking treatment under salt stress. The GA3 content increased significantly by 30.78% and 9.46%, respectively. The CTK content increased significantly by 39.57% and 59.45%, respectively.

### 4.11. Effect of Exogenous GR24 Immersion on Endogenous Hormone Balance in the Radicle and Germ Under Salt Stress

As shown in Table 5, the ratio of IAA/ABA and CTK/ABA in the radicle and germ decreased significantly under salt stress, and the decrease in the radicle was greater than that noted in the germ. The ratio of GA3/ABA to SL/ABA was also significantly reduced. Compared to CK, the ratios of IAA/ABA, CTK/ABA, GA3/ABA, and SL/ABA in the radicle and germ under salt stress were significantly reduced by 97.12% and 79.38%, 210.56% and 171.80%, 103.86% and 120.69%, and 126.14% and 64.52%, respectively. On the contrary, exogenous GR24 immersion treatment regulated hormonal balance. Compared to salt stress, the ratios of IAA/ABA, CTK/ABA, GA3/ABA, and SL/ABA in radicle and germ after exogenous GR24 soaking treatment under salt stress increased significantly by 51.97%, and 51.72%, 64.49% and 72.69%, 58.73% and 39.58%, and 75.60% and 75.82%, respectively.

## 5. Discussion

### 5.1. Exogenous GR24 Improved the Germination and Seedling Growth Ability of Rice Seeds Under Salt Stress

Seed germination is an important biological process that determines plant life cycles and crop production [36]. Under salt stress, seed germination was inhibited due to difficulty in absorbing water [37]. GR24 effectively mitigated salt stress-induced developmental retardation and oxidative damage by improving stress tolerance in plants [38]. It was found that low concentrations of GR24 significantly increased the number and length of the tips of lateral roots of oilseed rape [39]. In this study, the exogenous GR24 soaking treatment effectively alleviated the inhibition of rice seed germination under salt stress. The current study demonstrated that GR24 increased the germination rate, germination potential, germination index, radicle and germ length, dry and fresh weight, and number of lateral roots (Table 2 and Table 3; Figure 2). Our results are consistent with that of Li et al. [25], who reported that exogenous GR24 promoted the germination rate, radicle length and lateral root count of cucumber seeds under salt stress. Exogenous GR24 positively regulated the root and crown length of rice [40]. These results showed that exogenous GR24 soaking treatment alleviated the inhibition of salt stress on rice seed germination.

### 5.2. Exogenous GR24 Enhanced the Metabolism of Storage Substances During Seed Germination Under Salt Stress

The energy supply during the germination stage of the seed comes from the decomposition of the substances stored in the seed [41]. In a suitable external environment, the seeds can be transformed, and the stored substrates utilized, by initiating a series of internal enzymatic reactions [42]. Starch is the main storage form of sugar in seeds [43]. Starch was hydrolyzed into soluble sugar by amylase, and α-amylase activity was positively correlated with seed germination and seedling growth [44,45]. Soluble sugars and soluble proteins are osmoregulators and plant nutrients [46,47]. In our study, the catabolic capacity of starch decreased due to the decrease in amylase activity under salt stress. We speculate that it might be due to ionic toxicity caused by salt stress, which inactivates amylase [48]. Interestingly, after the application of exogenous GR24 soaking treatment, the amylase activities during seed germination under salt stress were all significantly higher than that of the salt stress treatment (Figure 6) and more soluble sugars and soluble proteins accumulated (Figure 4), which led to an increase in the rate of material conversion (Figure 3) and accelerated the capacity of starch catabolism (Figure 5). These results are similar to that of Amri et al. [27] and Huang et al. [47]. Current results suggested that exogenous GR24 plays an important role in accelerating the catabolism of storage material during seed germination in response to salt stress.

### 5.3. Exogenous GR24 Improves Antioxidant Capacity During Rice Seed Germination Under Salt Stress

Salt stress induces oxidative damage and leads to the loss of membrane integrity, and a change in MDA content is one of the important indicators of the degree of oxidative damage to cell membranes [49]. Previous studies have shown that exogenous GR24 attenuates the inhibition of heat stress on lupin seed germination by reducing membrane lipid peroxidation [50]. In this study, exogenous GR24 significantly reduced the MDA content in germinating seeds under salt stress after immersion treatment (Figure 7G), suggesting that GR24 could alleviate the oxidative damage caused by salt stress during seed germination by reducing the MDA content. Numerous studies have pointed out that GR24 regulates ROS homeostasis by increasing antioxidant enzyme activity and antioxidant content [51]. Our results found that SOD, POD, CAT, and APX activities were significantly higher during seed germination after exogenous GR24 imbibition treatment under salt stress (Figure 7A–D). This is consistent with the findings of Parisa et al. [52]. In addition, ASA and GSH contents were also significantly increased (Figure 7E,F), suggesting that exogenous GR24 accelerated the regeneration of ASA and GSH when the seeds were germinated under salt stress. The same findings were achieved when GR24 was exogenously applied to *Ajwain* [53], *melon* [23], *ornamental sunflower* [51] and *barley* [54]. This suggests that exogenous GR24 is able to influence the antioxidant system to alleviate oxidative stress induced by salt stress during seed germination.

### 5.4. Exogenous GR24 Regulates the Endogenous Hormone Balance in Embryonic Roots and Germs of Rice Seeds Under Salt Stress

Endogenous hormones play a direct or indirect regulatory role in the germination of grains [55,56]. Adverse stress usually disrupts the balance of endogenous plant hormones [57]. Daviere et al. [58] reported that increased salinity leads to enhanced ABA biosynthesis in plants. The results of Ma et al. [59] indicated that exogenous GR24 application increased the content of endogenous hormones and indirectly co-responded to salinity stress. Tian et al. [60] confirmed that exogenous GR24 treatment resulted in significant increases in IAA and CTK content and a significant decrease in ABA content in tobacco. In the present study, exogenous GR24 application under salt stress significantly decreased ABA content and increased SLs, IAA, GA3, and CTK content in the radicle and germ (Table 4). This is similar to the findings of Liu et al. [61]. Changes in the proportions of endogenous hormones reflect the integrated regulation of plants by hormones [62]. Ni et al. [39] showed that exogenous SLs promote lateral root growth in oilseed rape through interactions with other hormones. In this study, salt stress significantly reduced the IAA/ABA and CTK/ABA ratios of the radicle and germ, and the decrease in the radicle was greater than the decrease in the germ, indicating that the effect of salt stress on the radicle was greater than that on the germ (Table 5). However, exogenous GR24 treatment mitigated this phenomenon (Table 5), and the ratios of GA3/ABA and SL/ABA increased substantially. Our results are similar to the findings of Min et al. [63]. The above results indicated that exogenous GR24 dipping can regulate the content of endogenous hormones in the embryonic root and germ. This treatment regulates the hormonal balance of the embryonic root and germ and reduces the salt toxicity.

## 6. Conclusions

**Figure 8:**
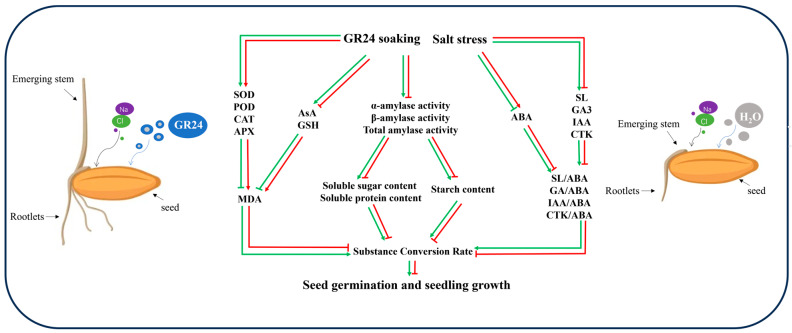
Exogenous GR24 soaking promotes seed germination and seedling growth by improving the catabolism of seed storage material and the balance of endogenous hormones. Green color indicates GR24+ salt stress; red color indicates salt stress. Lines with arrowheads represent promotion, and lines with a blunt end that lack an arrowhead represent inhibition.

Salt stress inhibited rice seed germination, and soaking rice seeds in GR24 at a concentration of 1.2 µmol L^−1^ could effectively alleviate the effects of salt stress on rice seed germination and seedling growth (Figure 8). Exogenous GR24-soaked seeds under salt stress accelerated starch catabolism by increasing amylase and antioxidant enzyme activity in the seeds, thus accumulating more soluble proteins, soluble sugars, and antioxidants. This led to increased material conversion and accelerated starch catabolism, which in turn promoted seed germination and seedling growth. Exogenous GR24 attenuated the inhibition of salt stress on rice seed germination and seedling growth and improved the salt tolerance of rice. The results of this study further elucidated the potential mechanism by which exogenous GR24 alleviated salt stress.

## Figures and Tables

**Table 1:** Effects of soaking seeds in different concentrations of GR24 on the radicle and germ lengths of rice.

Sampling Area	Treatment Number	Sampling Time		
		96 h	144 h	192 h
Plumule length (mm)	CK	11.52 ± 0.16452 e	36.41 ± 0.72701 d	41.28 ± 0.47814 d
	T1	11.71 ± 0.17285 de	37.89 ± 0.43677 cd	48.66 ± 1.37511 b
	T2	12.28 ± 0.28 cd	40.7 ± 0.59067 b	47.82 ± 0.47465 b
	T3	17.94 ± 0.32118 a	43.57 ± 0.36149 a	52.38 ± 0.58115 a
	T4	13.92 ± 0.23324 b	39.27 ± 0.51209 bc	45.58 ± 0.56506 c
	T5	12.51 ± 0.15452 c	36.5 ± 0.59367 d	44.42 ± 0.80523 c
Radicle length (mm)	CK	28.25 ± 0.46482 e	53.31 ± 1.48132 e	57.44 ± 0.92294 d
	T1	30.93 ± 0.23525 d	54.41 ± 0.45251 cd	56.04 ± 0.80955 d
	T2	32.45 ± 0.50996 c	58.72 ± 0.37706 b	61.84 ± 0.7123 c
	T3	37.21 ± 0.29153 a	64.63 ± 0.41421 a	70.44 ± 0.59576 a
	T4	33.78 ± 0.27031 b	60.67 ± 0.2757 b	65.03 ± 1.06082 b
	T5	31.44 ± 0.2997 cd	56.13 ± 0.83334 c	60.52 ± 1.65387 c

Note: CK: water soaking + water treatment; T1: 0.3 μmol L^−1^ GR24 soaking + water treatment; T2: 0.6 μmol L^−1^ GR24 seed soaking + water treatment; T3: 1.2 μmol L^−1^ GR24 seed soaking + water treatment; T4: 2.4 μmol L^−1^ GR24 soaking + water treatment; T5: 4.8 μmol L^−1^ GR24 soaking + water treatment. Values are means ± SD of three replicates. Different letters in the data columns indicate significant differences (*p* < 0.05) according to Duncan’s test.

**Table 2:** Effect of exogenous GR24 imbibition on the seed germination rate, germination potential, and germination index under salt stress.

Treatment Number	Germination Potential (%)	Germination Rate (%)	Germination Index (%)
CK	83.98 ± 1.472 a	93.33 ± 1.5784 a	13.33 ± 0.2255 a
G	92.12 ± 0.8612 a	97.68 ± 0.7653 a	13.95 ± 0.1093 a
N	48.59 ± 13.32 b	77.97 ± 4.3218 b	11.14 ± 0.6045 b
NG	75.83 ± 3.3298 a	92.46 ± 1.2524 a	13.21 ± 0.1789 a

Note: CK: Clear water + clear water treatment; G: 1.2 µmol L^−1^ GR24 dipped seed + clear water treatment; N: clear water dipped seed + 80 mmol L^−1^ NaCl treatment; NG: 1.2 µmol L^−1^ GR24 dipped seed + 80 mmol L^−1^ NaCl treatment. Values are means ± SD of three replicates. Different letters in the data columns indicate significant differences (*p* < 0.05) according to Duncan’s test.

**Table 3:** Effects of exogenous GR24 soaking on seed germ fresh weight, radicle fresh weight, germ dry weight, radicle dry weight, and lateral root number under salt stress.

Treatment Number	Fresh Weight of Germ (mg)	Fresh Weight of Radicle (mg)	Dry Weight of Germ (mg)	Dry Weight of Radicle (mg)	Number of Lateral Roots (Strips)
CK	14.57 ± 0.27327 b	18.98 ± 0.20859 b	3.62 ± 0.10414 b	1.42 ± 0.07717 b	4.1 ± 0.100 b
G	18.81 ± 0.23591 a	24.67 ± 0.34158 a	4.69 ± 0.0526 a	1.77 ± 0.08699 a	6.0 ± 0.2582 a
N	12.03 ± 0.23383 d	12.98 ± 0.34474 d	2.51 ± 0.06574 c	1.00 ± 0.06498 c	2.6 ± 0.1633 c
NG	13.22 ± 0.24712 c	17.14 ± 0.30155 c	3.51 ± 0.07219 b	1.36 ± 0.07024 b	3.5 ± 0.1000 b

Note: CK: Clear water + clear water treatment; G: 1.2 µmol L^−1^ GR24 dipped seed + clear water treatment; N: clear water dipped seed + 80 mmol L^−1^ NaCl treatment; NG: 1.2 µmol L^−1^ GR24 dipped seed + 80 mmol L^−1^ NaCl treatment. Values are means ± SD of three replicates. Different letters in the data columns indicate significant differences (*p* < 0.05) according to Duncan’s test.

**Table 4:** Effect of exogenous GR24 imbibition on endogenous hormone content in the radicle and germ under salt stress.

Sampling Area	Treatment	SLs	IAA	GA3	CTK	ABA
Plumule	CK	1453.41 ± 7.54 d	47.72 ± 0.99 c	365.88 ± 5.86 a	232.67 ± 6.52 c	570.89 ± 11.53 c
	G	4732.55 ± 26.93 a	75.27 ± 0.75 a	374.83 ± 1.82 a	391.22 ± 3.32 a	456.73 ± 4.77 d
	N	1519.30 ± 14.05 c	45.81 ± 0.85 c	287.21 ± 7.80 c	147.40 ± 5.61 d	662.14 ± 6.89 a
	NG	4244.56 ± 13.46 b	63.90 ± 0.93 b	317.23 ± 1.94 b	363.46 ± 6.00 b	668.21 ± 5.46 b
Radicle	CK	2111.10 ± 59.23 c	66.31 ± 0.31 b	321.98 ± 0.84 c	338.1 ± 7.42 a	610.32 ± 3.61 b
	G	4537.04 ± 18.20 a	90.59 ± 2.05 a	406.10 ± 5.40 a	324.93 ± 6.29 a	421.80 ± 5.31 d
	N	1485.73 ± 50.81 d	53.36 ± 0.63 c	247.42 ± 1.11 d	172.71 ± 7.25 c	968.06 ± 4.85 a
	NG	3824.96 ± 35.76 b	65.30 ± 0.36 b	357.45 ± 4.62 b	285.79 ± 2.97 b	569.09 ± 4.36 c

Note: CK: Clear water + clear water treatment; G: 1.2 µmol L^−1^ GR24 dipped seed + clear water treatment; N: clear water dipped seed + 80 mmol L^−1^ NaCl treatment; NG: 1.2 µmol L^−1^ GR24 dipped seed + 80 mmol L^−1^ NaCl treatment. Values are means ± SD of three replicates. Different letters in the data columns indicate significant differences (*p* < 0.05) according to Duncan’s test.

**Table 5:** Effect of exogenous GR24 seed immersion on endogenous hormone balance in the radicle and germ under salt stress.

Sampling Area	Treatment	IAA/ABA	CTK/ABA	GA3/ABA	SL/ABA
Plumule	CK	0.08 ± 0.0002 c	0.41 ± 0.0036 c	0.64 ± 0.0028 b	2.55 ± 0.0405 c
	G	0.16 ± 0.0003 a	0.86 ± 0.0029 a	0.82 ± 0.0066 a	10.36 ± 0.0557 a
	N	0.05 ± 0.0006 d	0.15 ± 0.005 d	0.29 ± 0.0063 d	1.55 ± 0.0243 d
	NG	0.1 ± 0.0007 b	0.55 ± 0.0052 b	0.48 ± 0.0012 c	6.41 ± 0.0327 b
Radicle	CK	0.11 ± 0.0002 c	0.55 ± 0.009 b	0.53 ± 0.0019 c	3.46 ± 0.0769 c
	G	0.21 ± 0.0026 a	0.77 ± 0.0052 a	0.96 ± 0.0014 a	10.76 ± 0.1042 a
	N	0.06 ± 0.0004 d	0.18 ± 0.0067 d	0.26 ± 0.0009 d	1.53 ± 0.047 d
	NG	0.11 ± 0.0004 b	0.5 ± 0.0027 c	0.63 ± 0.0051 b	6.27 ± 0.0211 b

Note: CK: Clear water + clear water treatment; G: 1.2 µmol L^−1^ GR24 dipped seed + clear water treatment; N: clear water dipped seed + 80 mmol L^−1^ NaCl treatment; NG: 1.2 µmol L^−1^ GR24 dipped seed + 80 mmol L^−1^ NaCl treatment. Values are means ± SD of three replicates. Different letters in the data columns indicate significant differences (*p* < 0.05) according to Duncan’s test.

## Data Availability

Data may be obtained through the corresponding author upon reasonable request. The data presented in this study are available on reasonable request from the corresponding author due to that the related research are continuing.
